# SARS-CoV-2 shedding, infectivity, and evolution in an immunocompromised adult patient

**DOI:** 10.1590/S1678-9946202466028

**Published:** 2024-05-13

**Authors:** Maria Cassia Mendes-Correa, Fábio de Rose Ghilardi, Matias Chiarastelli Salomão, Lucy Santos Villas-Boas, Anderson Vicente de Paula, Heuder Gustavo Oliveira Paiao, Antonio Charlys da Costa, Tânia Regina Tozetto-Mendoza, Wilton Freire, Flavia Cristina Silva Sales, Ingra Morales Claro, Ester Cerdeira Sabino, Nuno Rodrigues Faria, Steven Sol Witkin

**Affiliations:** 1 Universidade de São Paulo, Faculdade de Medicina, Instituto de Medicina Tropical de São Paulo (LIM-52), São Paulo, São Paulo, Brazil; 2 Universidade de São Paulo, Faculdade de Medicina, Instituto de Medicina Tropical de São Paulo (LIM-46), São Paulo, São Paulo, Brazil; 3 Hospital 9 de Julho, São Paulo, São Paulo, Brazil; 4 Universidade de São Paulo, Faculdade de Medicina, Hospital das Clínicas, São Paulo, São Paulo, Brazil; 5 Imperial College, Department of Infectious Disease Epidemiology, London, United Kindgom; 6 University of Oxford, Department of Zoology, Oxford, United Kindgom; 7 Weill Cornell Medicine, Department of Obstetrics and Gynecology, New York, New York, USA

**Keywords:** SARS-CoV-2, Cell culture, Evolution, Immunodeficiency, Shedding

## Abstract

This study aimed to provide further insight into the evolutionary dynamics of SARS-CoV-2 by analyzing the case of a 40-year-old man who had previously undergone autologous hematopoietic stem cell transplantation due to a diffuse large B-cell lymphoma. He developed a persistent SARS-CoV-2 infection lasting at least 218 days and did not manifest a humoral immune response to the virus during this follow-up period. Whole-genome sequencing and viral cultures confirmed a persistent infection with a replication-positive virus that had undergone genetic variation for at least 196 days after symptom onset.

## INTRODUCTION

Chronic infection of SARS-CoV-2 in immunocompromised individuals has been associated with intra-host viral evolution and immune escape^1-3^. However, data on replication potential, shedding, and viral diversity in long-term persistent infections in immunocompromised individuals remain scarce^1-3^. This report describes a unique case of SARS-CoV-2 viral dynamics and viability in different clinical specimens over a prolonged period in an immunocompromised host, whose replication-competent virus accrued an extensive number of mutations consistent with intra-host viral genomic evolution.

## CASE REPORT

This is a case of persistent SARS-CoV-2 infection in a 40-year-old man with a history of autologous hematopoietic stem cell transplantation (HSCT) for diffuse large B-cell lymphoma. On September 3, 2020 (day 0 of the disease), six months after HSCT (March 5, 2020), while on maintenance therapy with acyclovir, sulfamethoxazole, trimethoprim, glucocorticoids, and cyclo-phosphamide, the patient began to experience fever (37.8 °C), myalgia, and headache. On September 9, he sought medical assistance, underwent blood and swab tests, and was positive for SARS-CoV-2 by nasopharyngeal reverse transcription polymerase chain reaction (RT-PCR). A chest computed tomography (CT) scan revealed interstitial pneumonia restricted to the basal lung areas. The patient was discharged with oral antibiotics (amoxicillin/clavulanate plus azithromycin). A worsening of his symptoms on September 12 led to his return to the hospital, where he was admitted as an inpatient. During this first hospitalization, he received corticosteroids (prednisone) 0.5 mg/kg and a five-day course of ceftriaxone and azithromycin.

His clinical status and laboratory outcomes improved, and he was discharged on September 15. On September 18, he noticed new signs of dyspnea with fatigue and chest pain and complained of fever, although this was not measured. This clinical worsening led to a second hospitalization on September 21, which lasted until December 29. This prolonged stay was due to a relapse of his interstitial pneumopathy, with worsening clinical condition, lymphopenia, and low monocyte count. The patient also developed several bacterial infections related to this prolonged hospitalization.

Due to his severe condition, on October 2, the patient was treated with plasma from individuals who had recovered from COVID 19 (convalescent plasma). The procedure was uneventful, but the patient’s condition worsened and he required orotracheal intubation on October 6, as well as increased vasoactive drugs. He also received broad-spectrum antibiotics (linezolid and meropenem) and antifungal therapy (micafungin), but his condition worsened.

On October 26, a bronchoalveolar lavage (BAL) revealed that the patient had pneumonia due to *Klebsiella sp.* and *Stenotrophomonas maltophilia*, which were diagnosed by polymerase chain reaction (PCR). Despite all these clinical complications, his situation improved and he was discharged from the hospital on December 22.

On January 10, the patient developed fever and respiratory distress, and on January 15, he was readmitted to the hospital. At that time, his nasopharyngeal RT-PCR was still positive for SARS-CoV-2, and because of the respiratory decline, which raised concerns of fibrosing pneumonia, he also received intravenous immunoglobulin for one day, as well as daily antibiotics, while on corticosteroids. As his nasopharyngeal RT-PCR remained positive, a second infusion of convalescent plasma was administered on February 12. This led to an increased lymphocyte count and clinical improvement. The patient was discharged from the hospital on March 4.

Due to the patient’s impaired immunological status, persistent symptoms and prolonged positive RT-PCR result, it was decided to investigate the replicative capacity of his SARS-CoV-2 infection. From January 21 to April 9, blood, urine, saliva, and nasopharyngeal and anal swab samples were collected for analysis at weekly intervals.

## MATERIALS AND METHODS

From January 21 to April 9, 2021, blood, urine, saliva, and nasopharyngeal and anal swabs were collected weekly. Swab samples from the nasopharynx, collected at the hospital from September 9 to January 15, were sent to the Virology Laboratory of the Instituto de Medicina Tropical, Sao Paulo, Brazil. All samples collected were sent for viral identification, isolation, and serological analyses. RNA extraction was performed using the QIAamp viral RNA kit according to the manufacturer’s instructions. Quantitative assays (SARS-CoV-2 N or E gene) for SARS-CoV-2 were performed according to protocols adapted with primers and probes for the RT-PCR assay, as previously described^4^.

### Virus isolation

Viral culture of SARS-CoV-2 was conducted in a biosafety level 3 facility at the Virology Laboratory of the Instituto de Medicina Tropical de Sao Paulo, Sao Paulo State, Brazil, as previously described^4^.

### Virus Neutralization Test (VNT)

The presence of anti-SARS-CoV-2 antibodies was assessed using a cytopathic effect (CPE)-based virus neutralization assay. SARS-CoV-2 (EPI_ISL_1557222) was added to 96-well microtiter plates containing 5 × 10^4^ Vero cells/mL. This CPE VNT was applied as previously described by Villas-Boas *et al*.^5^.

### SARS-COV-2 genomic sequencing and molecular analysis

**Figure 1:**
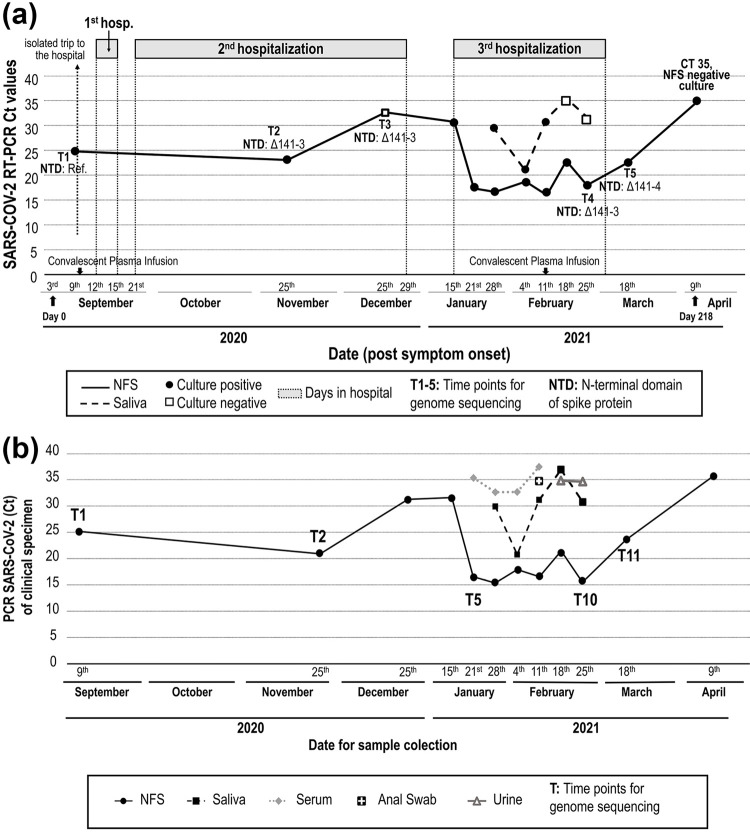
A) Timeline of SARS-CoV-2 molecular detection and genome sequencing from NFS and saliva samples (September 2020 to April 2021); B) Timeline of SARS-CoV-2 molecular detection by PCR from different clinical specimens and samples (September 2020 to April 2021).

The SARS-CoV-2 genomes from September 9 (T1), November 25 (T2), January 21 (T5), February 25 (T10) and March 18 (T11) were sequenced by multiplex PCR as described previously^6^ (Figure 1A). The available open COVID-19 sequencing and bioinformatics protocols developed by the ARTIC network were used as described elsewhere^7^. Reads were mapped against the reference sequence Wuhan-Hu-1 (GenBank Accession Number MN908947) and low coverage regions were masked with N characters. Genomes were classified using the Pango lineage nomenclature system^8^ and maximum likelihood (ML) phylogenetic analysis was performed using complete reference genomes IQtree v2^9^. A maximum likelihood tree was constructed using a nucleotide sequence alignment that included all complete genomes of B.1.1.28 sequences of SARS-CoV-2 from Brazil available in GISAID, collected before March 18, 2021. The consensus sequences generated in this study are available in GISAID under the IDs EPI_ISL_1857098, EPI_ISL_1857094, EPI_ISL_1857095, EPI_ISL_1857096, and EPI_ISL_1857097.

### Ethics

This study was approved by the local research ethics committee (CONEP, protocol Nº CAAE: 30419320.7.0000.0068, April 18, 2020) and the subjects involved provided written informed consent.

## RESULTS

All NFS samples collected from September 9 to April 9 were positive for SARS-CoV-2, a period of 218 days of continuous virus shedding. Samples collected after this date were negative for SARS-CoV-2 RNA (Table 1, Figures 1A and B). Five of the eight saliva samples collected from January 21 to April 9 were RNA negative. Four of the eight serum samples collected in the same period were positive for viral RNA. In addition, two of eight urine samples and one of eight anal swabs were positive for viral RNA (Figure 1B). Of the 44 clinical samples sent for virus isolation, 12 were positive after the second passage: nine from NFS and three from saliva (Table 1, Figure 1A). Replicating virus was detected in NFS or saliva samples for up to 196 days.

**Table 1:** Weekly molecular detection of SARS-CoV-2 from different clinical specimens and samples (September 9, 2020 to April 9, 2021).

Date of sample collection	Clinical specimen	PCR SARS-CoV-2 (Ct) of the clinical specimen		GS	CPE after the 1st passage in culture	PCR SARS-CoV-2 (Ct) after the 2^nd^ passage in culture		Final result of viral isolation (number of culture passages)
	
		Gene E	Gene S			Gene E	Gene S	
September 9	**NFS**	24.8	25.2	**T1**	Positive	29.6	26.5	**Positive (2)**
November 25	**NFS**	21.1	21.1	**T2**	Positive	26	28	**Positive (2)**
December 25	**NFS**	32.6	30		Negative	ND	ND	Negative (2)
January 15	**NFS**	31.5	31.7		Positive	35,93	ND	**Positive (2)**
January 21	**NFS**	16.6 35.3	16.9 35.5	**T5**	Positive	19.9 ND	ND ND	**Positive (2)**
	Serum							
January 28	**NFS**	14.9	15.2		Positive	12.5	11.6	**Positive (2)**
	**Saliva**	30.0	30		Positive	33	32	**Positive (2)**
	Serum	32.7	ND		Negative	ND	ND	Negative (2)
February 4	**NFS**	18.5	17.5		Positive	22	21	**Positive (2)**
	**Saliva**	20.8	21.0		Positive	25	24	**Positive (2)**
	Serum	32.7	32.8		Negative	ND	ND	Negative (2)
February 11	**NFS**	16,5	16.9		Positive	16	15	**Positive (2)**
	**Saliva**	31.2	31.3		Positive	34,9	33	**Positive (2)**
	Anal Swab	34.8	ND		Negative	ND	ND	Negative (2)
	Serum	37.2	38.7		Negative	ND	ND	Negative (2)
February 18	**NFS**	21.0	22.1		Positive	23	21.9	**Positive (3)**
	Saliva	37.0	ND		Negative	ND	ND	Negative (2)
	Urine	34.9	ND		Negative	ND	ND	Negative (2)
February 25	**NFS** Saliva Urine	15.7	15.6	**T10**	Positive	19	18	**Positive (3)**
	Saliva	31.0	30.7		Positive	ND	ND	Negative (2)
	Urine	34.7	ND		Positive	ND	ND	Negative (2)
March 18	**NFS**	23.8	23.6	**T11**	Negative	ND	ND	Negative (2)
April 9	**NFS**	35.1	36.4		Negative	ND	ND	Negative (2)

ND = Not Detected; NFS = Nasopharyngeal swabs; GS = Genome Sequencing; Ct = cycle threshold.

Sera collected on 10 occasions beginning on January 21 were consistently negative for neutralizing antibodies, suggesting acquisition of mutations that confer resistance to neutralizing antibodies^10^.

**Figure 2:**
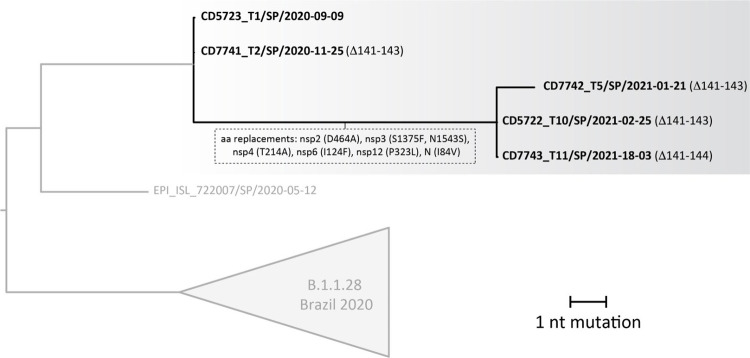
ML tree constructed using nucleotide sequence alignments including the complete genome of B.1.1.28 sequences of SARS-CoV-2 from Brazil available in GISAID with collection date <= March 18, 2021.

Virus genomes from samples collected on September 9 (T1), November 25 (T2), January 21 (T5), February 25 (T10), and March 18 (T11) were generated using a well-described multiplex PCR approach (Table 1). The assembled viral genomes achieved 75% to 98% genome coverage with a depth of at least 20x reads and were all classified as belonging to the B.1.128 lineage, which originated in Brazil^11^. The phylogenetic tree estimated with all B.1.1.28 genomes available up to T11 (*n*=1,300) indicated that the patient’s viral sequences clustered monophyletically with maximum statistical support (phylogenetic bootstrap support = 100%), a scenario consistent with long-term persistent infection of SARS-CoV-2 in a single individual (Figure 2).The closest available viral sequence was from Sao Paulo (GISAID ID: EPI_ISL_722007), consistent with local acquisition of the primary infection (the patient reported no travel history). The samples had five cluster-defining mutations (three synonymous and two nonsynonymous). One of these missense mutations affected two proteins as it was located in an overlapping region coding for both nucleoprotein and ORF9b products. Eight additional mutations were acquired from T2 to T5, seven of which were nonsynonymous (Figure 2). Most nonsynonymous mutations were retained until the last time point and were located in genes coding for non-structural proteins within the ORF1ab, which represents > 70% of the SARS-CoV-2 genome. Interestingly, at all-time points after T1, the viral strains had a deletion corresponding to three amino acids (L141-; G142-; V143-; Δ141-143) in the spike protein gene at the N-terminal domain of the S1 subunit (NTD-S1), distal to the receptor-binding site. At the last time point (T11), an additional deletion was present at residue 144 (Y144-; Δ141-144).

## DISCUSSION

In this study, we reported persistent SARS-CoV-2 shedding of infectious viruses in NFS and saliva samples collected from an immunocompromised individual over a period of 196 days. Virus genome sequences collected >6 months apart confirmed prolonged intra-host evolution and revealed different mutations, including a convergent 4-amino-acid deletion (Δ141-144) in the N-terminal domain (NTD) of S1 (RDR2 region) altering virus antigenicity^10^.

Our phylogenetic analysis and long-term shedding of infectious virus, confirmed by isolation in Vero cells, revealed independent acquisition of a replication-competent, antigenically distinct Δ141-144 variant. High-frequency variants originating from individuals with prolonged shedding can spread and be selected at the population level, highlighting public health concerns regarding clinical management of immunocompromised individuals, as recently described^12^.

We detected several amino acid deletions within the spike protein. The Δ141-143 in-frame 3-amino-acid deletion in the N-terminal domain (NTD) was accrued after the first infusion of convalescent plasma, between the first and second time points (7–22 days after symptoms onset). This deletion was maintained until the last day of sample collection. At some point between T10 and T11, the virus acquired an additional adjacent deletion in the NTD, Δ144. Based on structural studies, the S1 and S2 subunits of the spike protein mediate receptor binding and membrane fusion and form the bulbous head and stalk region^13^. Although this region is thought to be conformationally variable, changes within it likely impact the binding of proteins to the cellular receptor^14^. Interestingly, deletions in NTD are not uncommon and have been repeatedly observed by other authors, both in immunocompromised individuals with prolonged shedding and in immunocompetent patients, as well as in viral strains belonging to distinct lineages^10,15^. The convergent NTD deletion observed here and elsewhere is highly suggestive of viral adaptation in response to convergent selective pressures. This is supported by the failure of virus neutralization by antibody 4A8, which targets the NTD region in *in vitro* tests^10^.

Several cases of prolonged SARS-CoV-2 shedding, infectivity and evolution in immunocompromised patients have been reported in the literature^1,2,15-20^. However, to the best of our knowledge, this is the first study to report SARS-CoV-2 viral dynamics and viability in different clinical specimens over a prolonged period in an immunocompromised host. Most studies investigating SARS-CoV-2 infectivity and evolution have included either mainly respiratory samples or different biological samples, but only analyzed them for a short period^1,2,15-20^.

Our findings may have implications for the management of SARS-CoV-2 in long-term chronically infected individuals in community and/or health care settings. Although SARS-CoV-2 RNA was detected in most of the clinical specimens analyzed, including nasal secretions, saliva, serum, urine and anal samples, the importance of viral transmission from these last three sources seems to be at most marginally relevant to clinical practice. Although we were only able to detect replication-competent viruses in nasal secretions and saliva, we cannot rule out the possibility that this may partially be due to differences in viral titer in the various samples or to the presence of replication inhibitors. Nevertheless, our data suggest that considerations related to patient isolation should focus on viral presence in respiratory secretions and saliva in symptomatic patients.

A limitation of our study is the fact that we were unable to test the index patient’s contacts. Genome sequencing of viral isolates from clinical samples collected 196 days apart allowed us to observe changes in the SARS-CoV-2 genome over this extended period. Studies investigating whether viral strains with the recurrent deletions associated with immune escape observed here can be effectively transmitted at the population level are urgently needed. Implementation of contact tracing, especially for infections with longer generation intervals such as those described here, and continuous surveillance of recent SARS-CoV-2 cases in Brazil will help control community transmission of SARS-CoV-2 variants with altered epidemiological characteristics.

## CONCLUSION

In conclusion, this study shows that viable and replication-competent SARS-CoV-2 virus can be recovered from pharyngeal mucosa and saliva at prolonged intervals in immunocompromised patients. The present observations may be relevant for further refinement of prevention and transmission protocols.

## References

[1] Tarhini H, Recoing A, Bridier-Nahmias A, Rahi M, Lambert C, Martres P (2021). Long-term severe acute respiratory syndrome Coronavirus 2 (SARS-CoV-2) infectiousness among three immunocompromised patients: from prolonged viral shedding to SARS-CoV-2 superinfection. J Infect Dis.

[2] Sonnleitner ST, Prelog M, Sonnleitner S, Hinterbichler E, Halbfurter H, Kopecky DB (2022). Cumulative SARS-CoV-2 mutations and corresponding changes in immunity in an immunocompromised patient indicate viral evolution within the host. Nat Commun.

[3] Wang Y, Wang D, Zhang L, Sun W, Zhang Z, Chen W (2021). Intra-host variation and evolutionary dynamics of SARS-CoV-2 populations in COVID-19 patients. Genome Med.

[4] Mendes-Correa MC, Salomão MC, Ghilardi F, Tozetto-Mendoza TR, Villas-Boas LS, Paula AV (2023). SARS-CoV-2 Detection and culture in different biological specimens from immunocompetent and immunosuppressed COVID-19 patients infected with two different viral strains. Viruses.

[5] Villas-Boas LS, Paula AV, Silva AR, Paiao HG, Tozetto-Mendoza TR, Manuli ER (2022). Absence of neutralizing antibodies against the Omicron SARS-CoV-2 variant in convalescent sera from individuals infected with the ancestral SARS-CoV-2 virus or its Gamma variant. Clinics (Sao Paulo).

[6] Jesus JG, Sacchi C, Candido DS, Claro IM, Sales FC, Manuli ER (2020). Importation and early local transmission of COVID-19 in Brazil, 2020. Rev Inst Med Trop Sao Paulo.

[7] Lambisia AW, Mohammed KS, Makori TO, Ndwiga L, Mburu MW, Morobe JM (2022). Optimization of the SARS-CoV-2 ARTIC Network V4 primers and whole genome sequencing protocol. Front Med (Lausanne).

[8] Rambaut A, Holmes EC, O'Toole A, Hill V, McCrone JT, Ruis C (2020). A dynamic nomenclature proposal for SARS-CoV-2 lineages to assist genomic epidemiology. Nat Microbiol.

[9] Trifinopoulos J, Nguyen LT, von Haeseler A, Minh BQ (2016). W-IQ-TREE: a fast online phylogenetic tool for maximum likelihood analysis. Nucleic Acids Res.

[10] McCarthy KR, Rennick LJ, Nambulli S, Robinson-McCarthy LR, Bain WG, Haidar G (2021). Recurrent deletions in the SARS-CoV-2 spike glycoprotein drive antibody escape. Science.

[11] Candido DS, Claro IM, Jesus JG, Souza WM, Moreira FR, Dellicour S (2020). Evolution and epidemic spread of SARS-CoV-2 in Brazil. Science.

[12] Lee JS, Yun KW, Jeong H, Kim B, Kim MJ, Park JH (2022). SARS-CoV-2 shedding dynamics and transmission in immunosuppressed patients. Virulence.

[13] Tang T, Bidon M, Jaimes JA, Whittaker GR, Daniel S (2020). Coronavirus membrane fusion mechanism offers a potential target for antiviral development. Antiviral Res.

[14] Walls AC, Park YJ, Tortorici MA, Wall A, McGuire AT, Veesler D (2020). Structure, function, and antigenicity of the SARS-CoV-2 spike glycoprotein. Cell.

[15] Avanzato VA, Matson MJ, Seifert SN, Pryce R, Williamson BN, Anzick SL (2020). Case study: prolonged infectious SARS-CoV-2 shedding from an asymptomatic immunocompromised individual with cancer. Cell.

[16] Choi B, Choudhary MC, Regan J, Sparks JA, Padera RF, Qiu X (2020). Persistence and evolution of SARS-CoV-2 in an immunocompromised host. N Engl J Med.

[17] Sung A, Bailey AL, Stewart HB, McDonald D, Wallace MA, Peacock K (2022). Isolation of SARS-CoV-2 in viral cell culture in immunocompromised patients with persistently positive RT-PCR results. Front Cell Infect Microbiol.

[18] Kim MC, Cui C, Shin KR, Bae JY, Kweon OJ, Lee MK (2021). Duration of culturable SARS-CoV-2 in hospitalized patients with Covid-19. N Engl J Med.

[19] Cunha MP, Vilela AP, Molina CV, Acuña SM, Muxel SM, Barroso VM (2021). Atypical prolonged viral shedding with intra-host SARS-CoV-2 evolution in a mildly affected symptomatic patient. Front Med (Lausanne).

[20] Nussenblatt V, Roder AE, Das S, de Wit E, Youn JH, Banakis S (2022). Yearlong COVID-19 infection reveals within-host evolution of SARS-CoV-2 in a patient with B-cell depletion. J Infect Dis.

